# Laryngology and Rhinology

**Published:** 1893-03

**Authors:** B. J. Baron


### LARYNGOLOGY AND RHINOLOGY.

All who are in the habit of examining the larynx must have noticed how very variable in position, shape, and size the epiglottis is, and how frequently it is in contact with parts of the tongue which normally ought not to be touched by it. The symptoms of this are a feeling of tickling with spasmodic cough or vomiting, and a sensation of fulness in the throat which is very disagreeable; if the lingual tonsil is enlarged along with a thickened, misplaced, and congested epiglottis, all these symptoms are exaggerated. The lingual tonsil can be subjected to the same treatment as the faucial tonsil quite safely, but no one proposed to deal with the epiglottis until Rice [1] ventured to remove a piece of the offending cartilage with a pair of long-handled scissors. Bleeding was free, but was easily checked, and no serious trouble from inflammatory swelling supervened. The operation was successful in every way.

The influence of faulty voice-production in causing disease of the throat, and in preventing cure of inflammatory and catarrhal conditions of the pharynx and larynx in people whose occupations compel the use of the vocal apparatus other than in conversation—*e.g.* clergymen, teachers in schools, barristers, public reciters, singers, and actors—is well known to all specialists in the treatment of throat and nose disease, but it is practically ignored by others of our profession. This most important question was freely discussed at the Nottingham meeting of the British Medical Association,[2] when Sandford, Lennox Browne, and Hunt showed conclusively that stammering, chronic pharyngitis, loss of the singing voice, and the so-called "clergyman's sore throat," are all caused, and their cure rendered practically impossible, by faulty voice-production.

Although we have such excellent teachers of singing in Bristol, we have no teacher of general voice-production; and one's work in the treatment of throat ailments that directly arise from ignorance of these matters is thereby greatly hampered.

As it seems probable that influenza may make its appearance amongst us once more, it may be worth while to record Beverley

[1] *New York Med. Journ.*, Apr. 9, 1892.
[2] Report in *Journ. of Laryn., Rhin., and Otol.*, Sept., 1892.

Robinson's formula[1] for the sudden inflammation of the muscles of the neck, with pain, redness, and rigidity, accompanied by throat troubles, that occur in that disease. Every two or three hours is to be taken one tablet containing half a grain of citrate of caffeine, one grain of phenacetine, and three grains of ammonium salicylate.

Papers on the ever interesting question of nasal neuroses are always appearing, and abstracts of a few of them will, we think, be of value.

Bosworth[2] reports 88 cases of asthma in which there was co-existent nasal disease of a turgescent character, and in which surgical interference for its relief was undertaken. The result of treatment was: 42 patients had no attack for at least twelve months after, 33 were improved (*i.e.* the attacks were less violent, and came at longer intervals), 2 were not improved, and in 11 cases no information could be obtained.

W. Spencer Watson[3] says that asthma was present only three times in some hundreds of cases of chronic hypertrophic rhinitis, and in one case in which it had lasted eighteen years a cure was effected by the removal of polypi and hypertrophied turbinates.

H. Drinkwater[4] relates two cases of asthma cured after removal of nasal polypi.

Musehold[5] has cured a case of Basedow's disease by removing the posterior half of the inferior turbinated body with the galvano-cautery snare. The headache which was present at once disappeared, the heart-beat became nearly normal in rate after seven days, and the thyroid enlargement disappeared in two months under the influence of mild continuous currents. The author believes that this disease is sometimes due to reflex nasal neurosis.

Donald Stewart and Bronner[6] thus summarise their suggestions for treatment of nasal neurosis:

1. Redundant tissue must be removed with saw, cutting forceps, snare, trephine, or galvano-cautery and caustic acids, according to its character, position, &c.

2. Alkaline and antiseptic douches are of value in getting parts into a normal condition.

3. General tonic treatment must be adopted.

I have succeeded in curing a fairly severe case of epilepsy by removing several polypi from the nose and cauterising the hypertrophied turbinates, &c.

Cases of croupous rhinitis are published from time to time, and doubtless the disease occurs more frequently than has been

[1] *New York Med. Journ.*, Apr. 16, 1892. [2] *Ibid.*, Mar. 26, 1892.

[3] *Lancet*, Sept. 10, 1892. [4] *Brit. Med. Journ*, Mar. 16, 1892.

[5] *Rev. de Laryn.*, July 15, 1892.

[6] *Journ. of Laryn., Rhin., and Otol.*, Sept., 1892.

hitherto suspected. The irritation, discharge, and blocked-up state of the nostril, and the finding, on examination, of tough membrane coating and adhering so closely to the turbinates and septum that detachment of it leaves a bleeding surface, reminding one of croupous exudation in the larynx, are very characteristic.

Middlemass Hunt[1] reports a case in which he freely removed the membrane, dusted the abraded surface with iodoform, and employed an antiseptic spray. In fifteen days the membrane ceased to form, and the patient soon got well.

Newcomb[2] uses injections of boracic acid solutions with success.

Sedziak employs tampons soaked in equal parts of balsam of Peru and glycerine, and applies an ointment of boracic acid and resorcin and insufflations of aristol.

I have found boracic acid solution freely used with a properly constructed nasal syringe, and an ointment of eucalyptol and iodoform, extremely and quickly useful in a well-marked case, which was accompanied by non-syphilitic keratitis and iritis.

B. J. BARON.

### Reviews of Books.

[1] *Journ. of Laryn. and Rhin.*, Dec., 1891.
[2] *New York Med. Journ.*, Sept. 12, 1891.

